# Effect of a Zr-Based Metal–Organic Framework Structure on the Properties of Its Composite with Polyaniline

**DOI:** 10.1021/acsami.3c03870

**Published:** 2023-05-04

**Authors:** Konstantin A. Milakin, Sonal Gupta, Libor Kobera, Andrii Mahun, Magdalena Konefał, Olga Kočková, Oumayma Taboubi, Zuzana Morávková, Jia Min Chin, Kamal Allahyarli, Patrycja Bober

**Affiliations:** † Institute of Macromolecular Chemistry, Czech Academy of Sciences, 162 00 Prague, Czech Republic; ‡ Department of Physical and Macromolecular Chemistry, Faculty of Science, Charles University, 128 40 Prague, Czech Republic; § Institute of Inorganic Chemistry-Functional Materials, University of Vienna, A-1090 Vienna, Austria

**Keywords:** polyaniline, metal−organic framework, grafting, NMR, electrochemical characterization

## Abstract

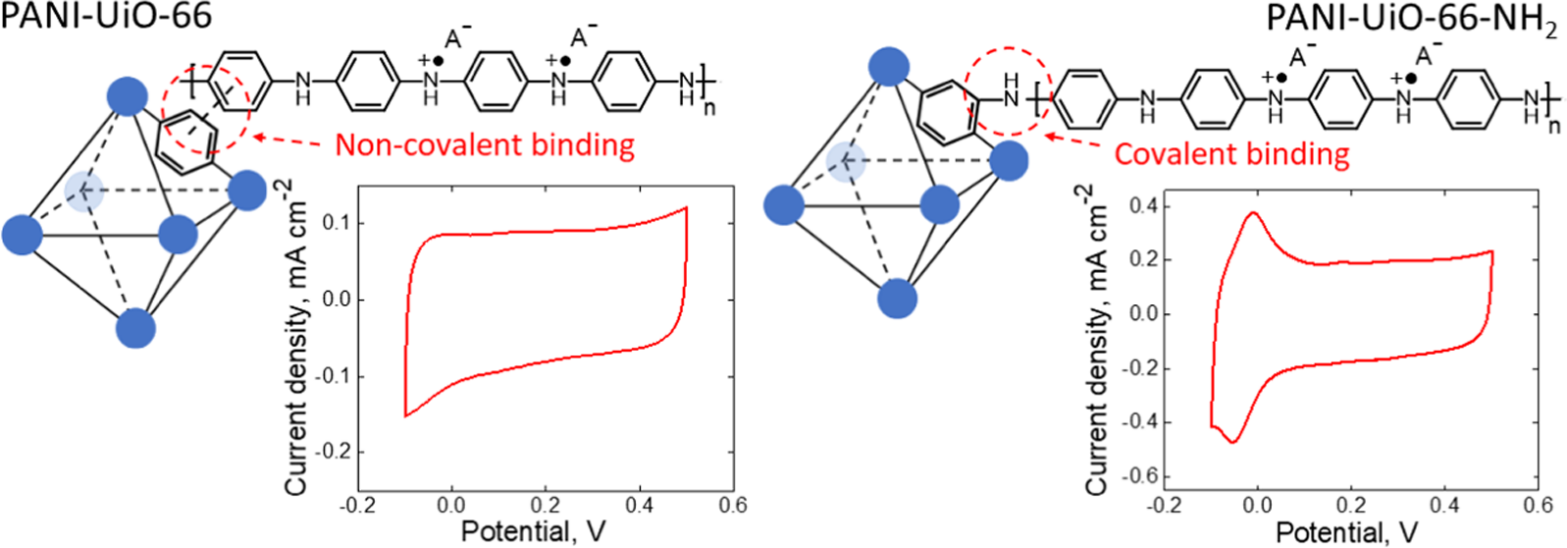

Composites of polyaniline (PANI) and Zr-based metal–organic frameworks (MOFs), UiO-66 and UiO-66-NH_2_, were synthesized by the oxidative polymerization of aniline in the presence of MOF templates with the MOF content in the resulting materials (78.2 and 86.7 wt %, respectively) close to the theoretical value (91.5 wt %). Scanning electron microscopy and transmission electron microscopy showed that the morphology of the composites was set by the morphology of the MOFs, whose structure was mostly preserved after the synthesis, based on the X-ray diffraction data. Vibrational and NMR spectroscopies pointed out that MOFs participate in the protonation of PANI and conducting polymer chains were grafted to amino groups of UiO-66-NH_2_. Unlike PANI-UiO-66, cyclic voltammograms of PANI-UiO-66-NH_2_ showed a well-resolved redox peak at around ≈0 V, pointing at the pseudocapacitive behavior. The gravimetric capacitance of PANI-UiO-66-NH_2_, normalized per mass of the active material, was also found to be higher compared to that of pristine PANI (79.8 and 50.5 F g^–1^, respectively, at 5 mV s^–1^). The introduction of MOFs into the composites with PANI significantly improved the cycling stability of the materials over 1000 cycles compared to the pristine conducting polymer, with the residual gravimetric capacitance being ≥100 and 77%, respectively. Thus, the electrochemical performance of the prepared PANI-MOF composites makes them attractive materials for application in energy storage.

## Introduction

Polyaniline (PANI) is one of the most studied conducting polymers with attractive properties and a wide range of possible applications,^1^ including sensors,^2−4^ actuators,^5,6^ supercapacitors,^7−9^ electrocatalysts,^10,11^ anticorrosion materials,^12,13^ etc. However, for most practical tasks, PANI is used as a part of various composite materials, which allows us to combine intrinsic properties of the components for achieving beneficial synergistic effects, improving the handling characteristics, and expanding potential application opportunities.^8,13^

Metal–organic frameworks (MOFs) are novel materials with a modular structure, which consists of metal cluster nodes bound by organic linkers.^14^ Due to their flexible composition and functionalization paired with porous structure and high specific surface area,^14^ they can be used for drug delivery,^15−17^ catalysis,^18−20^ sensing,^21−23^ or gas storage^24−26^ and can be attractive templates for PANI-based composites.^11,27,28^ However, they are considered to have relatively low thermal, hydrothermal, and chemical stability, which can hinder their applicability.^29^ Development of Zr-based MOFs, such as UiO-66 and its derivatives, showing superior thermal stability is a step toward overcoming the mentioned drawbacks.^29,30^ UiO-66, which consists of Zr_6_O_4_(OH)_4_ clusters bound by terephthalic acid ligands, and its derivative UiO-66-NH_2_ with 2-aminoterephthalic acid as a ligand^31^ have been successfully used as templates for the synthesis of PANI-based composites.^32−39^ PANI-UiO-66 or PANI-UiO-66-NH_2_ was prepared by either chemical^32−38^ or electrochemical^39^ approaches and used as sensors,^32,35^ adsorbents,^33,39^ and supercapacitor electrode materials.^36−38^ The most common chemical polymerization procedure included oxidative polymerization of aniline in the presence of the MOF dispersion.^32−34,37^ The resulting composite materials were found to have high specific surface area^32,34,37^ and excellent electrochemical performance.^32,37^ However, most of the published works do not study the effect of the MOF nature and functionalization on the structure and properties of the prepared materials, which is important for their further development and designing the products with desired characteristics. To the best of our knowledge, there is only one work^34^ that directly compares PANI-based composites synthesized in the presence of UiO-66 and UiO-66-NH_2_. Shanahan et al.^34^ reported that amino-functionalized UiO-66-NH_2_ showed better cohesion with PANI and higher chemical stability in the polymerization conditions compared to UiO-66. The structural differences, such as hindered PANI fiber formation, resulted in PANI-UiO-66-NH_2_ having lower conductivity than PANI-UiO-66. However, the mentioned work^34^ studied the materials with relatively low MOF content (aniline:MOF ratio during the synthesis varied from 1:1 to 3:1), which can partially mask the effect of MOF on the properties of the composite. Moreover, although the covalent grafting of PANI to amino groups of UiO-66-NH_2_ was mentioned in the literature, there was no direct evidence of it.^34,37^

Thus, in the present work, we have focused on studying the influence of the MOF nature and functionalization on the structure and properties of PANI-UiO-66 and PANI-UiO-66-NH_2_ composite materials. To ensure the maximum possible effect of MOF on the properties of the products, chemical polymerization was performed at the excess of MOF compared to the monomer. The difference in the chemical structures of the obtained composites was systematically studied by various spectroscopic methods, including solid-state NMR, in an attempt to directly show the grafting of PANI to amino groups of UiO-66-NH_2_ for the first time. A comparison of the electrochemical performances of PANI-UiO-66 and PANI-UiO-66-NH_2_ was conducted for assessing their potential applicability.

## Experimental Section

### Materials

Aniline hydrochloride (p.a., Penta, Czech Republic), ammonium peroxydisulfate (p.a., Lach-Ner, Czech Republic), terephthalic acid (>98% purity, Alfa Aesar, Germany), 2-aminoterephthalic acid (99% purity, Alfa Aesar, Germany), zirconium tetrachloride (>99.5% purity, Sigma-Aldrich, Germany), and Nafion 117 solution (∼5% in a mixture of lower aliphatic alcohols and water, Sigma-Aldrich) were used as received.

### Preparation of MOF

**Figure 1:**
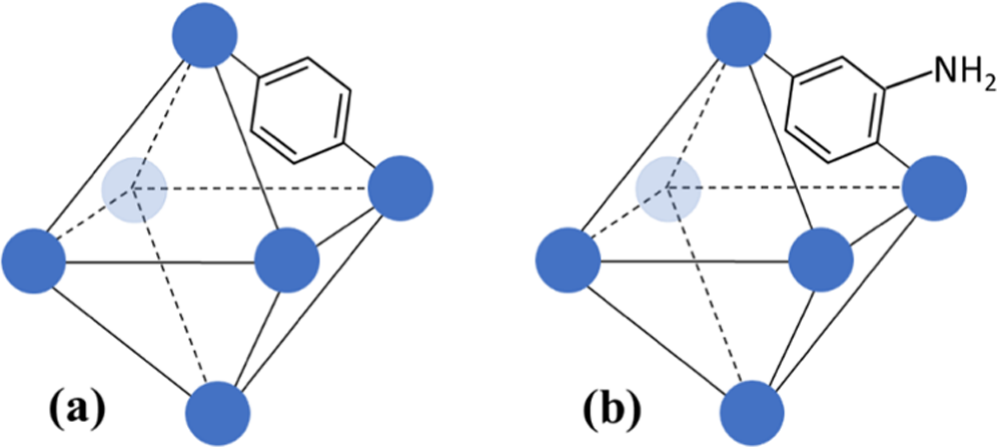
Schematic structures of (a) UiO-66 and (b) UiO-66-NH_2_, consisting of Zr_6_O_4_(OH)_4_ clusters (blue circles), connected with terephthalic acid or 2-aminoterephthalic acid linkers, respectively.

UiO-66 and UiO-66-NH_2_ MOFs were synthesized by the following procedure, in accordance with the method of Katz et al.^40^ ZrCl_4_ (0.54 mmol) was sonicated in a mixture of 5 mL of dimethylformamide (DMF) and 1 mL of concentrated HCl (12 M) for 20 min until fully dissolved. Then, the ligand, terephthalic acid or 2-aminoterephthalic acid (0.75 mmol), and 10 mL DMF were added to the mixture and it was sonicated for an additional 20 min before being heated at 80 °C overnight. The resulting solid was collected by centrifugation and washed with DMF (twice) and then with ethanol (twice). The samples were dried at 80 °C overnight. The schematic structures of MOFs, consisting of Zr_6_O_4_(OH)_4_ clusters, connected with terephthalic acid or 2-aminoterephthalic acid linkers, are shown in Figure 1.^31^

### Synthesis of PANI-MOF Composites

For the preparation of PANI-MOF composites, 3.5 mL of 0.02 M aniline hydrochloride solution in 0.01 M HCl was added to 0.082 g of MOF and left under stirring (500 rpm) for 1 h. Then, 3.5 mL of 0.025 M ammonium peroxydisulfate solution in 0.01 M HCl was added to the polymerization medium, and the reaction was left under stirring (500 rpm) overnight (23 h). The polymerization products were separated by centrifugation, washed with 0.01 M HCl and acetone, and left to dry in the air. Pristine PANI was synthesized in the absence of MOF by a similar procedure.

### Characterization

Determination of Zr in the materials was performed by the following procedure: the amount of the sample, adjusted according to the expected content of Zr (4–10 mg), was weighed into the glass vial and 1 mL of HNO_3_ (65%, p.a.) was added, followed by digestion in the Biotage Initiator microwave reactor. After the digestion, an aliquot of the digestion solution was diluted volumetrically with Milli-Q water (1:4) and measured by energy-dispersive X-ray fluorescence spectrometry using a SPECTRO Analytical Instruments SPECTRO XEPOS energy-dispersive X-ray fluorescence spectrometer, equipped with the silicon drift detector and the excitation system with a 50 W Pd anode X-ray tube. A standard solution of Zr (*c* = 1000 mg L^–1^) in the mixture of 5% HNO_3_ and 1% HF was used for Zr calibration. An aluminum oxide Barkla target was used for the quantitative analysis. The measurement time was 30 min. The chamber was flushed with He during the sample analyses. The Spectro Xepos software (TurboQuant method) was used for data analysis.

The morphology of the materials was studied using a Tescan MAIA3 scanning electron microscope (SEM) and an FEI Tecnai G2 Spirit transmission electron microscope (TEM).

X-ray diffraction (XRD) spectra were acquired using a GNR Analytical Instruments Explorer high-resolution diffractometer supplied with a one-dimensional Dectris Mythen 1K silicon strip detector. The Cu Kα anode (wavelength λ= 1.54 Å) operating with 40 kV and 30 mA and monochromatized with Ni Kβ filter was used. The data were obtained between 2θ = 5–50° with a step size of 0.1°. The exposure time at each step was 15 s. The degree of crystallinity was calculated according to the formula
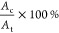
where *A*_c_ and *A*_t_ are the area of the crystalline peaks and the total area of the peaks, respectively.

Thermogravimetric analysis (TGA) was performed using a PerkinElmer Pyris 1 TGA thermogravimetric analyzer in the temperature range 30–800 °C at a heating rate of 10 °C min^–1^ in air.

Raman spectra were collected on a Renishaw inVia Reflex Raman spectrometer (Leica DM LM microscope; objective magnification 50×) with a diode 785 nm laser (holographic grating 1200 lines mm^–1^) and with a Thermo Nicolet 6700 Fourier transform infrared (FTIR) spectrometer with an FT Raman module NXR (Nd:YAG laser 1064 nm) in backscattering geometry, resolution 2 cm^–1^, and 1024 scans per spectrum on the samples pressed in potassium bromide pellets.

FTIR spectra in the region 4000–400 cm^–1^ were recorded using a Thermo Nicolet NEXUS 870 FTIR spectrometer (DTGS TEC detector; 64 scans; resolution 2 cm^–1^) in the transmission mode in potassium bromide pellets. The spectra were corrected for the carbon dioxide and humidity in the optical path.

^1^H and ^13^C solid-state NMR (ssNMR) spectra were recorded at 16.4 T using a Bruker AVANCE Neo NMR spectrometer. The 1.3 mm ultrafast magic-angle spinning (MAS) probe was used for the experiment at Larmor frequencies of ν(^1^H) = 700.302 MHz and ν(^13^C) = 176.110 MHz, respectively. ^1^H and ^13^C NMR isotropic chemical shifts were calibrated using α-glycine (^1^H: 3.5 ppm; splitting on −CH_2_– group; ^13^C: 176.03 ppm; carbonyl signal) as an external standard.^41^

The conductive PANI-based samples require the use of the very fast (VF/MAS) NMR approach.^42^ Therefore, the ^1^H very fast (VF) MAS NMR experiments were conducted at a MAS spinning speed of 60 kHz. The rotor synchronized spin-echo pulse sequence^43^ (π/2–*t*_1_–π–aq.) with one loop was used for all samples. The spectra were recorded using a 1.425 μs π/2 pulse with a recycle delay of 5 s and 16 scans. The two-dimensional ^1^H–^1^H SD/MAS NMR spectrum was recorded using nuclear Overhauser enhancement spectroscopy (NOESY)-type three-pulse sequence. The spectral width in both frequency dimensions was 20 kHz. The indirect detection period *t*_1_ consisted of 256 increments each made of eight scans. ^13^C VF/MAS NMR spectra were acquired using rotor synchronized spin-echo pulse sequence (π/2–*t*_1_–π–aq.) with one loop. The π/2 pulse with a length of 2.75 μs at 24.5 W was applied with a 1 s repetition delay and 80 000 scans. The rate of sample spinning was 60 kHz under MAS. Other samples were measured using the ^13^C CP/MAS NMR technique at a 30 kHz spinning speed with a 2 ms spin-lock. The repetition delay was 5 s, and for all ^13^C CP/MAS NMR experiments, the SPINAL 64 decoupling was used with 10k–40k scans. The sample was packed into a ZrO_2_ rotor and subsequently kept at room temperature. To compensate for frictional heating caused by the rotation of the sample, the NMR experiments were conducted under active cooling. The sample temperature was maintained at 300 K.^44^ All spectra and their fitting were processed using Top Spin 3.2 pl5 software package.

Electrochemical studies were performed on a Metrohm AUTOLAB PGSTAT302N using glassy carbon (diameter = 3 mm) as a working electrode, Ag/Ag^+^ wire as a pseudoreference, and Pt wire as a counter electrode. A 0.01 M HCl was used as a supporting electrolyte. For the deposition of the materials onto the working electrode, ∼4 mg of the material and ∼1 mg of carbon black were ground and dispersed uniformly in 1000 μL of a solution, comprising 590 μL of Milli-Q water, 400 μL of isopropanol, and 10 μL of Nafion 117 solution. Next, 1 μL of the dispersion was drop cast onto a glassy carbon electrode under an inert atmosphere.

The gravimetric capacitance was calculated using cyclic voltammograms (CV) at various scan rates (5–400 mV s^–1^) by the following equation
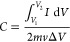
where ∫*I* d*V*, *v* (V s^–1^), and *m* (g) are the area under the CV curve, scan rate, and mass of the deposited material, respectively. Δ*V* is the potential window ranging from −0.1 to 0.5 V.

The study of the cycling stability of the materials was performed in 0.01 M HCl at 100 mV s^–1^ in the potential window from −0.1 to 0.5 V.

## Results and Discussion

### Preparation and Composition of PANI-MOF Composites

PANI-UiO-66 and PANI-UiO-66-NH_2_ composites were prepared by the oxidative polymerization of aniline in the presence of UiO-66 and UiO-66-NH_2_, respectively. It was noted that the reaction medium, containing the initial white dispersion of UiO-66-NH_2_, turned blue immediately after the addition of the oxidant, while the one with the UiO-66 dispersion remained initially unchanged and became darker after 30 min from the start of polymerization. After 23 h, both reaction mixtures were colored. As the obtained PANI-UiO-66-NH_2_ powder was gray, PANI-UiO-66 was brown. The observed difference in the polymerization kinetics can likely be attributed to the presence of the amino group in the structure of UiO-66-NH_2_, which can affect the aniline polymerization kinetics in a similar way as the addition of the other aromatic amines like *p*-phenylenediamine or benzidine.^45^

MOF fractions in the resulting PANI-MOF composites were recalculated from the Zr content measured by elemental analysis (Table 1). The calculated MOF content values for PANI-UiO-66 (78.2 wt %) and PANI-UiO-66-NH_2_ (87.6 wt %) were found to be close to the theoretical MOF content (91.5 wt %) in the composites, estimated using the expected theoretical PANI yield (0.84 g of PANI per 1 g of the monomer^46^). The lower fraction of UiO-66 in the corresponding composite might be attributed to its partial degradation during polymerization, which was pointed out by XRD (see the X-ray Diffraction section).

**Table 1:** Zr Content, Determined by Elemental Analysis, and Recalculated MOF Fraction in PANI-UiO-66 and PANI-UiO-66-NH_2_ Composites and Pristine MOFs

	Zr, wt %	MOF fraction, wt %
UiO-66	30.01	100
PANI-UiO-66	23.47	78.2
UiO-66-NH_2_	25.88	100
PANI-UiO-66-NH_2_	22.67	87.6

### Morphology

**Figure 2:**
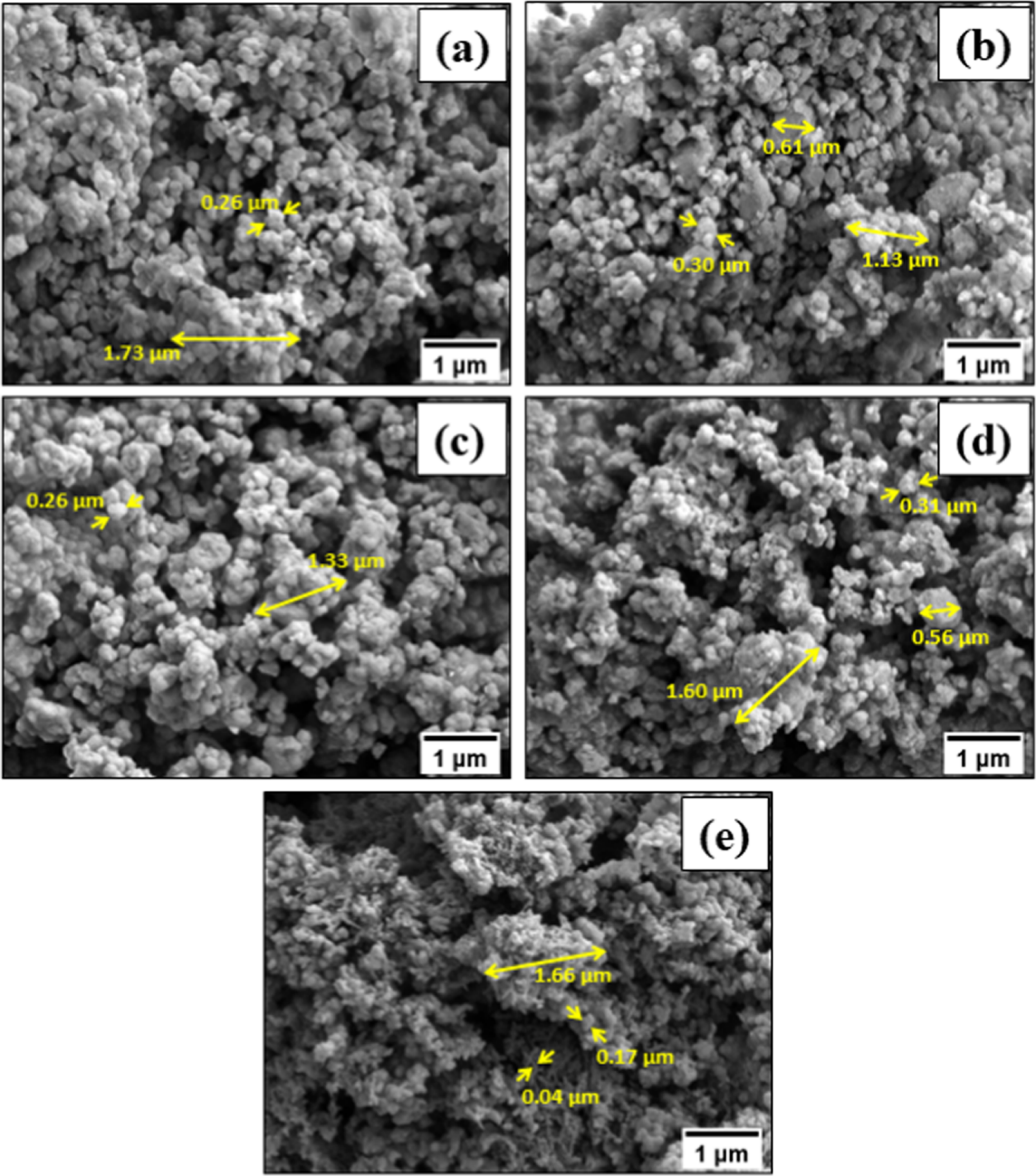
SEM images of pristine MOFs (a) UiO-66 and (b) UiO-66-NH_2_, composites (c) PANI-UiO-66 and (d) PANI-UiO-66-NH_2_, and (e) PANI synthesized in the absence of MOFs.

SEM images of the prepared PANI-UiO-66 and PANI-UiO-66-NH_2_ powders (Figure 2) show that their morphology is similar to the one of the initial MOF, although slightly more aggregated. The materials mostly consist of spherical particles with an average size of around 300 nm and aggregates reaching 1.7 μm. The morphology of PANI, prepared in the absence of the MOFs, is significantly different. It consists of significantly smaller irregularly shaped particles and aggregates (<1.7 μm), including globules (80–180 nm size) and one-dimensional structures (around 40 nm width). Therefore, SEM shows that the morphologies of the PANI-UiO-66 and PANI-UiO-66-NH_2_ composites are mainly determined by the morphology of the MOFs, with additional aggregation after polymerization.

**Figure 3:**
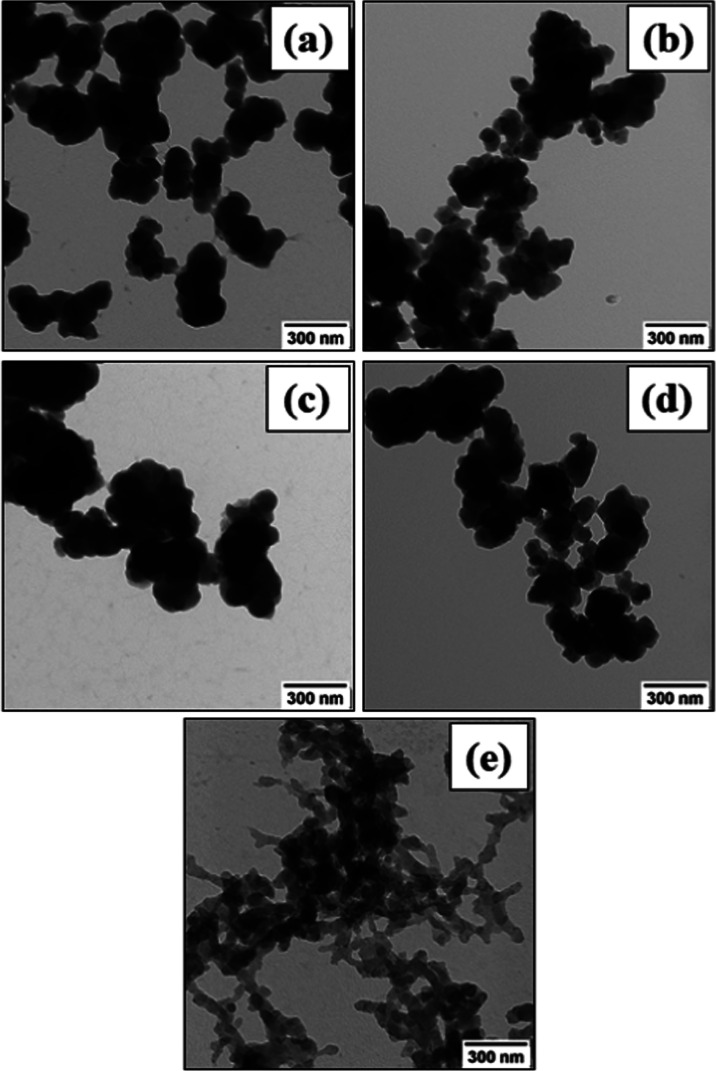
TEM images of pristine MOFs (a) UiO-66 and (b) UiO-66-NH_2_, composites (c) PANI-UiO-66 and (d) PANI-UiO-66-NH_2_, and (e) PANI synthesized in the absence of MOFs.

TEM images (Figure 3) confirm that the morphology of the prepared composites significantly differs from the morphology of pristine PANI. One-dimensional pristine PANI structures are clearly visible (Figure 3e), and it should also be noted that the observed morphology is different from the typical globular PANI, which can be obtained by the conventional IUPAC-recommended procedure.^47^ The difference is likely attributed to the use of the more diluted monomer solution, 0.01 M, in the present work, compared to the typically used ∼0.2 M.^46^ It was previously shown that the formation of one-dimensional structures is favorable when the polymerization proceeds in the diluted aqueous aniline solution.^48^ Moreover, TEM shows that no PANI structures were formed apart from the MOF particles, which points to the growth of PANI predominantly on the MOF surface.

### X-ray Diffraction

**Figure 4:**
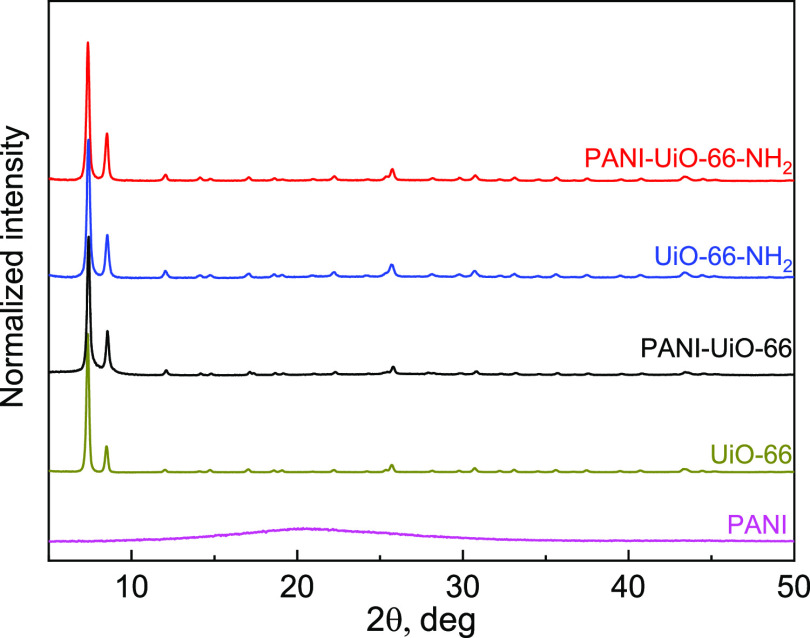
XRD patterns of pristine PANI, UiO-66, UiO-66-NH_2_, and PANI-MOF composites.

XRD analysis was additionally used to assess the MOF structural changes after the composite synthesis, and the XRD patterns of pristine PANI, MOFs, and PANI-MOF composites are presented in Figure 4. For pristine PANI, the very wide peak around 2Θ = 20.6° is observed, indicating its amorphous nature. Both pristine MOFs exhibit similar diffraction peaks at positions, characteristic for the UiO-66 and UiO-66-NH_2_ structures, reported in the literature,^49−51^ with a high degree of crystallinity (95 and 90% for UiO-66 and UiO-66-NH_2_, respectively). XRD patterns confirm that in both PANI-MOF composites, the MOF structure is preserved. The amorphous part, characteristic of PANI, was not present in the PANI-MOFs spectra, and it agrees with the previous results obtained for the PANI/iron(III) 1,3,5-benzenetricarboxylate composites.^33^ This observation can be attributed to either the low content of PANI or its homogeneous distribution in the material. For PANI-UiO-66-NH_2_ the XRD pattern is almost identical to pristine UiO-66-NH_2_, indicating that the MOF crystallinity in the composite is preserved (degree of crystallinity of 89% for PANI-UiO-66-NH_2_). However, in the case of PANI-UiO-66, the degree of crystallinity decreased to 85%. The sizes of the crystallites were calculated before and after PANI deposition by employing the Scherrer equation, according to the methodology presented in detail by Yot et al.^52^
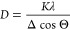
where the dimensionless shape factor *K* was taken equal to 0.9, wavelength λ = 1.5418 Å, the Bragg angle of the most intense diffraction peak (111) corresponding to 2θ ≈ 7.4° was used for Θ, while Δ is the full width at half-maximum (FWHM) of this peak. For UiO-66, the sizes of the crystallites decreased after PANI deposition from 68.4 to 66.9 nm, which, together with the loss of crystallinity, could suggest some partial degradation of the MOF structure. In contrast, for PANI-UiO-66-NH_2_, the sizes of the crystallites increased from 55.6 to 61.3 nm, which could be caused by the deposition of the PANI layer. The MOF structure can potentially be affected by two separate factors, originating from the preparation of the composites: the influence of the polymerization medium and the effect of deposited PANI. The reports on the stability of UiO-66 and UiO-66-NH_2_ in the acidic media, similar to the one, used in the present work for the polymerization (pH ≈ 2), are not conclusive. In the literature,^51,53,54^ based on qualitative XRD data, it was reported that no considerable changes in the MOF diffraction patterns (and, thus, the crystallinity) were observed in low-pH conditions. However, at the same time, qualitatively indistinguishable changes in the XRD patterns can still be accompanied by a decrease in the MOF specific surface area (shown at pH 0 for both UiO-66 and UiO-66-NH_2_).^51^ Moreover, for UiO-66, it was shown^54^ that treatment with acidic media (pH 1 and 3) can affect the MOF crystallite size. Depending on the MOF preparation procedure and origin, either a decrease or an increase in the crystallite size was observed.^54^ It is also known that the synthesis of the materials, where PANI can be intercalated in the substrate, can lead to the change of the substrate structure, such as its partial exfoliation.^55^ Thus, although the MOF structures are mostly preserved after the composite synthesis, the pH of the polymerization medium and the PANI layer deposition can both contribute to the observed changes in the XRD patterns.

### Thermogravimetric Analysis

**Figure 5:**
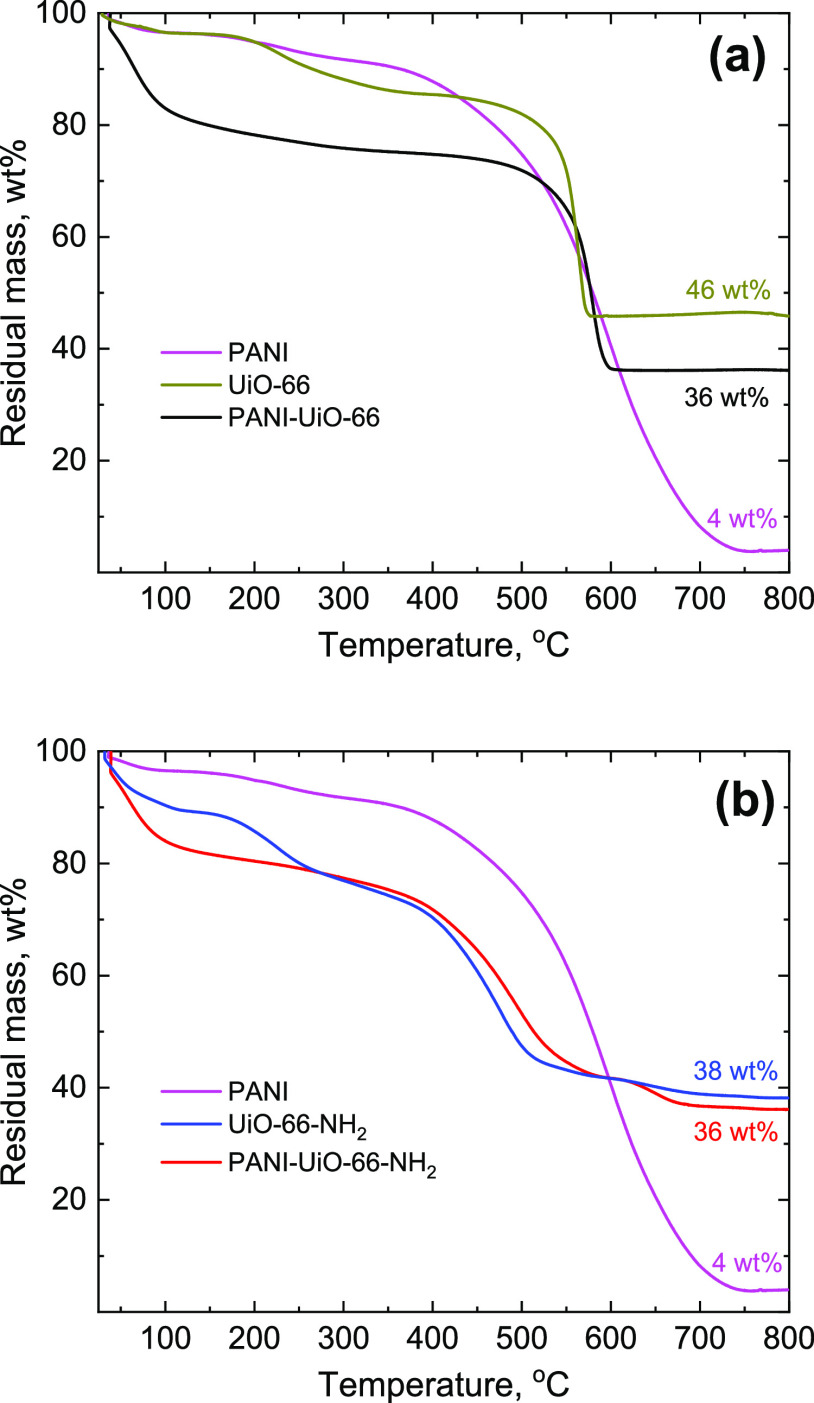
TGA curves (in air) of (a) PANI-UiO-66 and (b) PANI-UiO-66-NH_2_ composites in comparison with pristine PANI and pristine MOFs.

The thermal stability of PANI-UiO-66 and PANI-UiO-66-NH_2_ composites, pristine PANI, and MOFs were assessed by TGA. TGA (Figure 5) shows that UiO-66 has three main weight loss regions: <120, 170–400, and 450–580 °C, corresponding to the loss of physically adsorbed water, dehydroxylation of Zr nodes,^56^ and decomposition of terephthalic acid linkers in the MOF structure.^57^ The corresponding weight loss regions for UiO-66-NH_2_ are slightly shifted to the lower temperatures compared to UiO-66: <130, 160–280, and 380–520 °C. The residual weight in both cases corresponds to the formation of ZrO_2_: 46 wt % for UiO-66 and 38 wt % for UiO-66-NH_2_.^57,58^ Pristine PANI, synthesized in the absence of MOFs, also has three weight loss regions at <95, 150–300, and 380–750 °C, attributed to the elimination of water, dopant, and decomposition of PANI chains, respectively, with the last one being the most pronounced.^59^ In contrast to pristine MOFs, the polymer is almost fully decomposed at 750 °C with a residual weight of <4 wt %. PANI-MOF composites have two main weight loss regions, overlapping with those of the respective pristine MOFs: PANI-UiO-66 at <120 and 480–605 °C; PANI-UiO-66-NH_2_ at <120 and 360–600 °C. Therefore, it can be concluded that the thermal decomposition pattern of the composite materials is mainly determined by the MOF component rather than PANI. It should also be noted that the initial weight loss stage (<120 °C), connected with the elimination of physically adsorbed water, is more pronounced for the composites compared to the pristine MOFs, due to the synthesis being performed in aqueous media and water-adsorption capability of MOFs.^60^

### Spectroscopy

**Figure 6:**
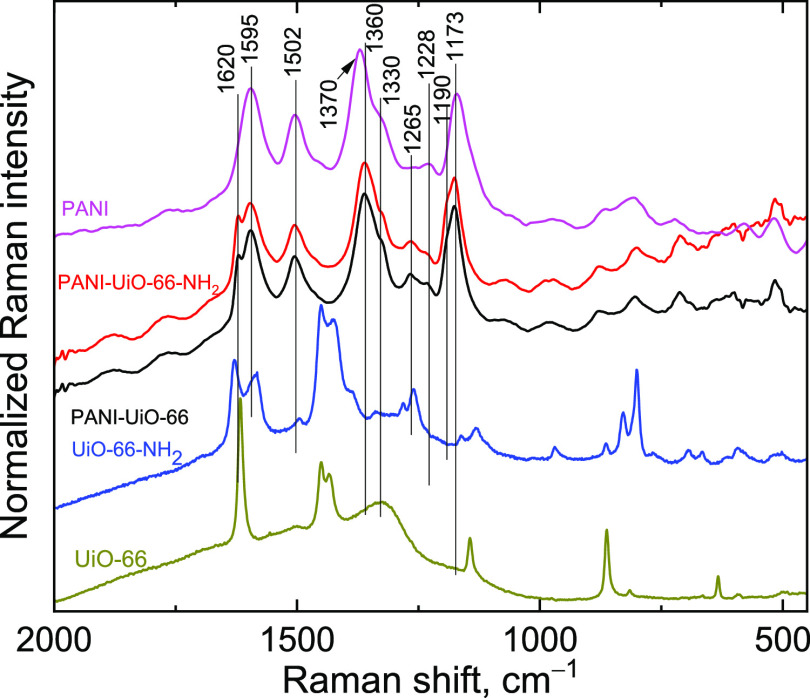
Raman spectra of the pristine MOFs (exc. 785 nm), PANI (exc. 1064 nm), and PANI-MOF composites (exc. 1064 nm) measured in KBr pellets.

The chemical structure of the prepared PANI-MOF composites was investigated by vibrational spectroscopy. Raman spectra (Figure 6) were measured using two excitation wavelengths of 785 and 1064 nm. The PANI-MOF composites spectra show fluorescence, when excited with wavelengths shorter than 1064 nm, whereas for pure MOFs, the best spectra were obtained with 785 nm excitation.

The Raman features of the MOFs are not observed in the spectra of the composites, as the signal from PANI is resonantly enhanced at 1064 nm. Therefore, Raman spectra are ideal for the discussion of the state of PANI in PANI-MOF composites. Pristine PANI displays Raman bands at 1595 cm^–1^ (ring stretching vibrations dominated by quinonoid and semiquinonoid rings), 1502 cm^–1^ (NH deformation), 1370 cm^–1^ (C–N^+•^ stretching in localized polaronic structures) with a shoulder at 1330 cm^–1^ (C–N^+•^ stretching in delocalized polaronic structures), 1265 cm^–1^ (C–N stretching on a quinonoid ring), 1228 cm^–1^ (C–N stretching), and 1173 cm^–1^ (C–H deformation on benzenoid and semiquinonoid rings).^61^

In the PANI-MOF composites, in comparison to the spectrum of pristine PANI, an additional band at 1620 cm^–1^ (ring stretching) was observed, the intensity of the band of C–N stretching next to a quinonoid structure at 1265 cm^–1^ increased, and the C–H deformation band had a shoulder at 1190 cm^–1^. These bands are related to benzoquinonoid structures.^61,62^ The C–N^+•^ stretching in localized polaronic structures also shifts from 1370 to 1360 cm^–1^ (toward weaker localization), which is connected with better chain organization. It may be caused by a shorter chain length.

**Figure 7:**
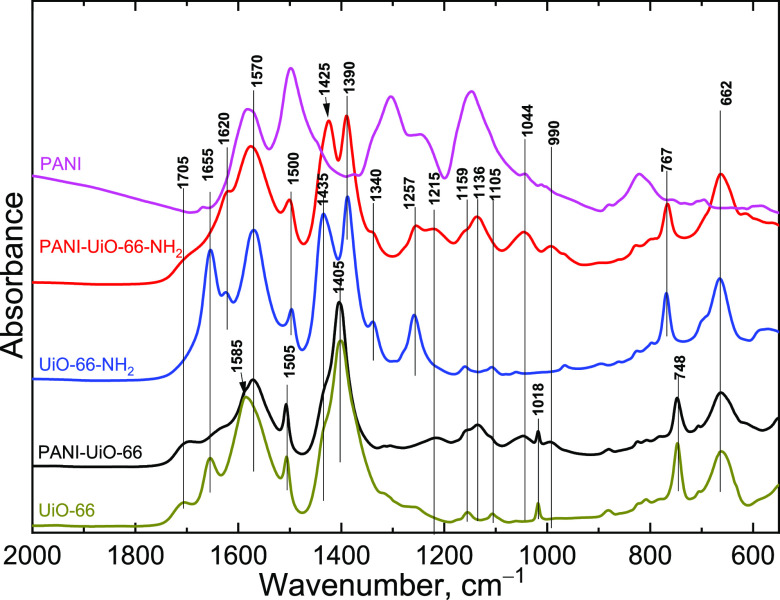
FTIR spectra of the PANI-MOF composites, pristine MOFs, and PANI measured in KBr pellets.

The FTIR spectra (Figure 7) reflect the MOF as a major component of the system. Pristine UiO-66 displays bands at 1705 cm^–1^ (C=O stretching in nondissociated acid groups), 1655 and 1585 cm^–1^ (carboxylate anion asymmetrical stretching), 1505 cm^–1^ (C=C stretching of the aromatic ring), 1435 and 1405 cm^–1^ (carboxylate anion symmetrical stretching), 1159, 1105, and 1018 cm^–1^ (in-plane C–H deformation), 748 cm^–1^ (out-of-plane C–H deformation), and 662 cm^–1^ (Zr–O stretching of the central Zr-cluster).^63−65^ For the amine-modified UiO-66-NH_2_, in contrast to UiO-66, amine group-related vibrations appear at 1620 cm^–1^ (in-plane deformation), 1340, and 1257 cm^–1^ (C–N stretching),^63,65^ carboxylate anion asymmetrical stretching shifts to 1570 cm^–1^, C=C stretching of the aromatic ring shifts to 1500 cm^–1^, carboxylate anion symmetrical stretching shifts to 1390 cm^–1^, and out-of-plane C–H deformation shifts to 767 cm^–1^. In addition, the C=O stretching in nondissociated acid groups disappears, pointing at better dissociation of the acid groups of UiO-66-NH_2_.

For the composites with PANI, the COO^–^ stretching area transforms to a single broad band at 1585/1570 cm^–1^, as the MOF acid groups protonate PANI. The position of this band’s maximum also correlates with a stretching of the quinonoid ring of aniline oxidation products. In the PANI-MOF composites, new bands appear at 990, 1044, 1136, and 1215 cm^–1^, which are probably related to sulfate,^66^ originating from ammonium peroxydisulfate. C–N stretching band of UiO-66-NH_2_ amino groups at 1257 cm^–1^ also decreases slightly, which can be due to the reaction of the NH_2_ groups with aniline. A small shoulder of carbonyl stretching of COOH groups is present in both composite spectra.

**Figure 8:**
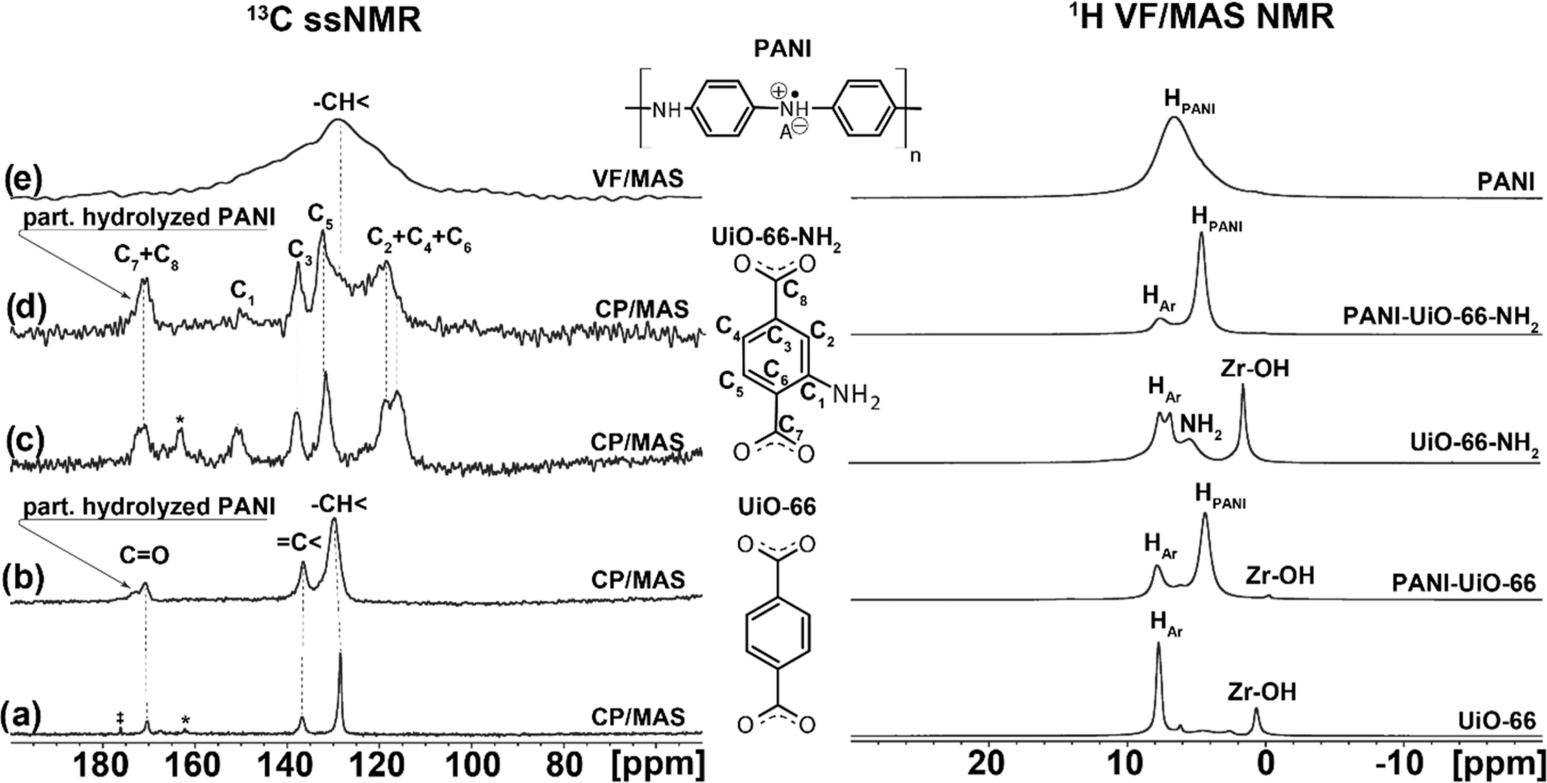
Experimental ^13^C ssNMR spectra (left-hand panel), schematic representation of the organic species (middle panel), and ^1^H VF/MAS NMR spectra (right-hand panel) of (a) UiO-66, (b) PANI-UiO-66, (c) UiO-66-NH_2_, (d) PANI-UiO-66-NH_2_, and (e) PANI, synthesized in the absence of MOFs. The peaks corresponding to residual solvent (DMF) and impurity are marked by * and ‡, respectively. The full-range experimental ^13^C ssNMR spectra in the original scale are depicted in the Supporting Information (Figure S1).

The ^1^H and ^13^C ssNMR spectroscopy was used to additionally directly investigate the structural features of PANI-UiO-66 and PANI-UiO-66-NH_2_ composites in comparison with the pristine components. Experimental ^1^H VF/MAS NMR and ^13^C ssNMR (VF/MAS and CP/MAS for PANI and MOFs, respectively) spectra of pristine materials (Figure 8a,c,e) were attributed based on the literature data^67−69^ and supported the proposed structures (Figure 8, middle panel). New signals, marked as H_PANI_, were observed in the ^1^H VF/MAS NMR spectra (Figure 8b,d right-hand panel), confirming the incorporation of PANI into MOFs in PANI-MOF composites. Moreover, the comparison of ^1^H VF/MAS NMR spectra of pristine MOFs and PANI-MOF composites, clearly indicates that Zr–OH groups interact with PANI chains. It could be attributed to the participation of MOF in the protonation of PANI, which agrees with the vibrational spectroscopy data, showing increased protonation of PANI in the composites compared to that in the pristine polymer, synthesized in the absence of MOFs. The corresponding ^13^C CP/MAS NMR spectra show that PANI in the PANI-MOF composites can be partially hydrolyzed,^70^ which is manifested by new carbonyl signals at ca. 173.5 ppm. Furthermore, according to a comparison of NMR patterns in Figure 8 (left-hand panel), ^13^C CP/MAS NMR spectra also indicate different behavior of PANI in the composites with different MOFs. In the case of PANI-UiO-66, the ^13^C CP/MAS NMR spectrum shows a significant broadening of the detected signals and a noticeable shift of the peak corresponding to −CH< aromatic carbons. The broadening of the NMR signals indicates immobilization of the respective moieties, and the change in position of the −CH< aromatic carbons peak suggests that they are involved in physical interaction. On the contrary, in the case of PANI-UiO-66-NH_2_, a broad −CH< signal, corresponding to PANI, overlaps the signals of aromatic carbons of the UiO-66-NH_2_ template. Moreover, the almost missing signal at 150.5 ppm (marked C_1_) clearly indicates that −NH_2_ groups, present in pristine UiO-66-NH_2_, are affected by the polymerization of aniline, which can likely be attributed to the grafting of PANI chains to the UiO-66-NH_2_ skeleton.

**Figure 9:**
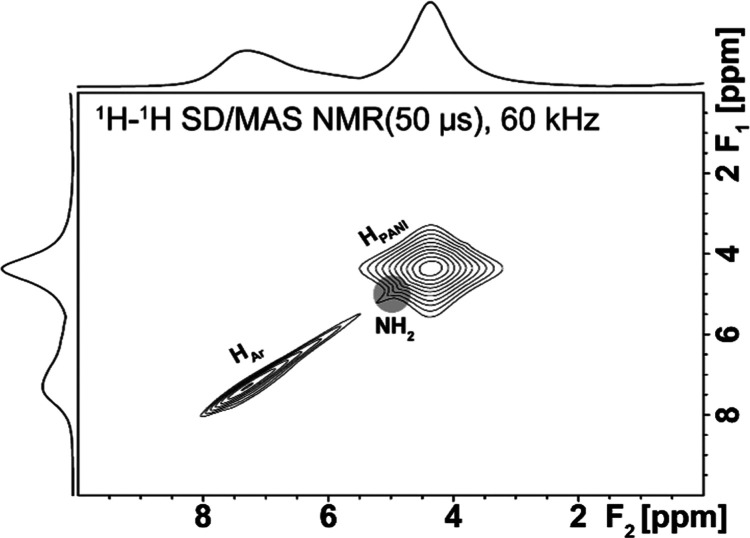
Experimental ^1^H–^1^H SD/MAS NMR of the PANI-UiO-66-NH_2_ system recorded with 50 μs mixing time. The highlighted signal in the gray circle corresponds to the residual −NH_2_ groups of pristine UiO-66-NH_2_ in the PANI-UiO-66-NH_2_ composite.

**Figure 10:**
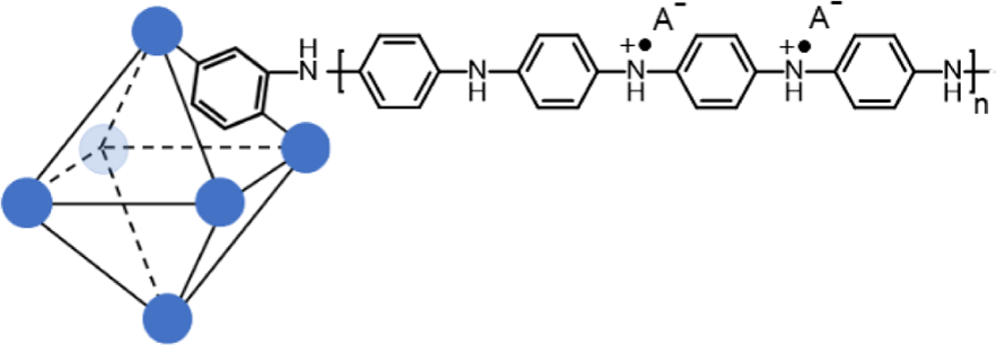
Schematic structure of the PANI-UiO-66-NH_2_ composite.

The confirmation of almost fully reacted −NH_2_ groups with PANI was additionally provided by the 2D ^1^H–^1^H SD/MAS NMR experiment (Figure 9). As can be seen from the provided spectrum, only a small residual amount of −NH_2_ groups of UiO-66-NH_2_ remains intact after the preparation of the PANI-UiO-66-NH_2_ composite, as shown by the highlighted signal in Figure 9. It corresponds well with the findings from the ^13^C CP/MAS NMR spectrum (Figure 8d, left-hand panel). Based on the presented results, the following structure of the PANI-UiO-66-NH_2_ composite, showing the grafting of PANI chains to MOF, can be proposed (Figure 10).

### Electrochemical Characterization

**Figure 11:**
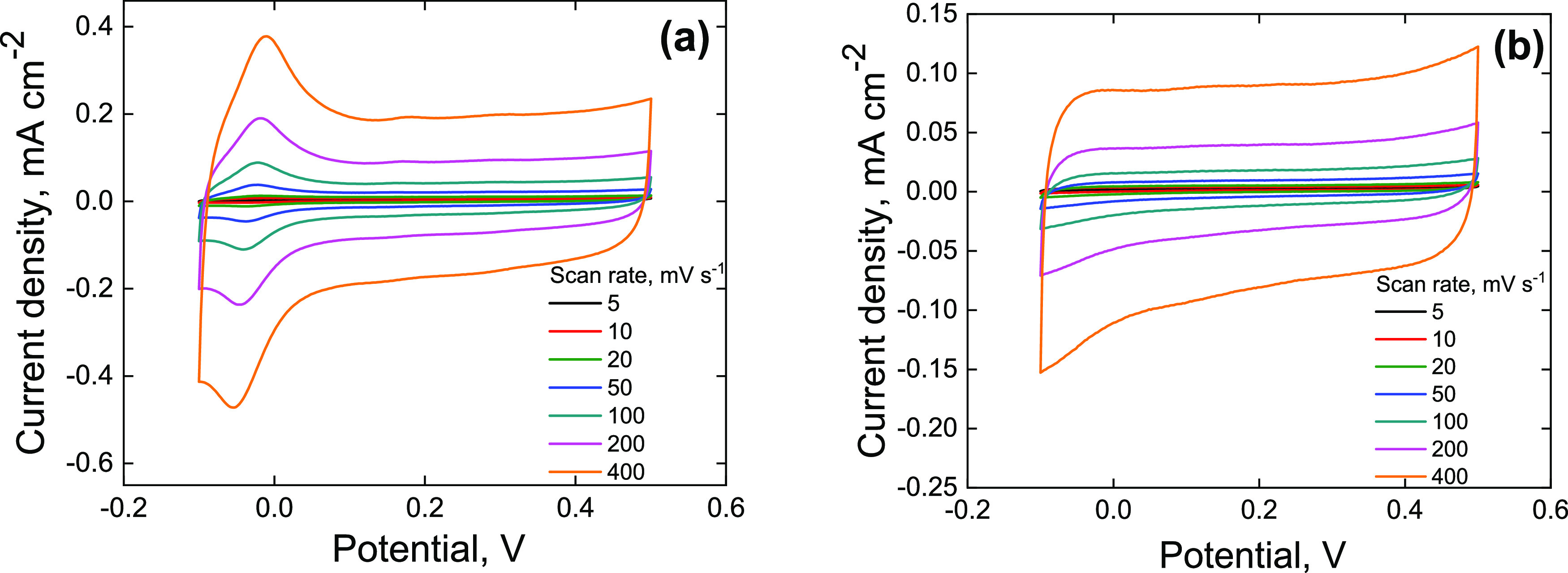
Cyclic voltammograms of (a) PANI-UiO-66-NH_2_ and (b) PANI-UiO-66, recorded at various scan rates in 0.01 M HCl.

The electrochemical properties of the PANI-MOF composites were assessed by cycling voltammetry. The results show that the nature of MOF significantly affects the voltammograms of the composites (Figure 11). The voltammogram of PANI-UiO-66 has a mostly rectangular shape, corresponding to the double-layer capacitive behavior, while the voltammogram of PANI-UiO-66-NH_2_ shows a well-resolved redox peak at around ≈0 V, pointing at a pseudocapacitive response contribution. Moreover, the voltammetric plots of both composites differ significantly from the voltammogram of pristine PANI (Figure S2), which can be attributed to the interaction between the components in the composites, suggested by vibrational spectroscopy and NMR. The gravimetric capacitance of PANI-UiO-NH_2_, normalized per mass of the active material (PANI), was also found to be slightly higher (79.8 F g^–1^ at 5 mV s^–1^) compared to the gravimetric capacitance of pristine PANI (50.5 F g^–1^ at 5 mV s^–1^). The highest obtained capacitance value for PANI-UiO-66 was 33 F g^–1^, measured at 5 mV s^–1^. The gravimetric capacitance values for the other scan rates are provided in the Supporting Information (Table S1).

**Figure 12:**
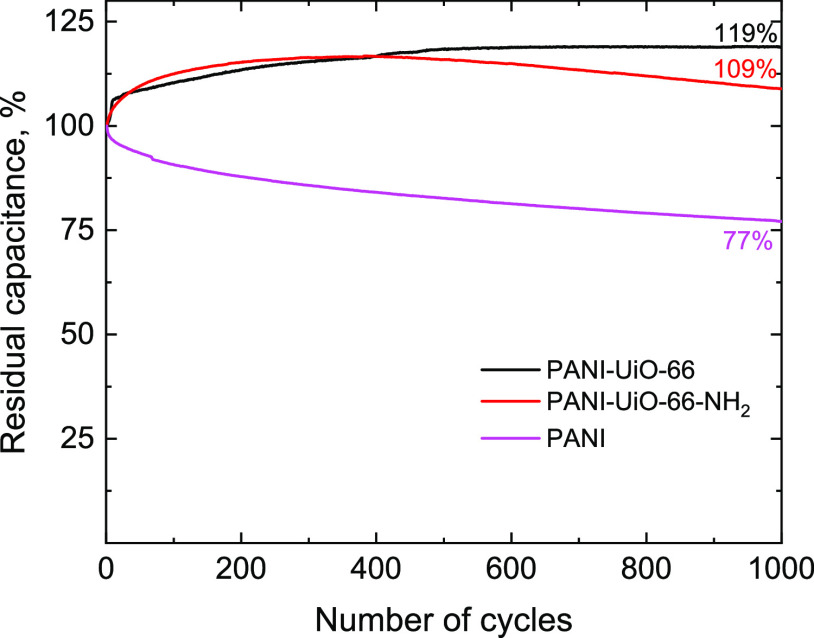
Cycling stability of pristine PANI, PANI-UiO-66-NH_2_, and PANI-UiO-66 composites in 0.01 M HCl at 100 mV s^–1^. The corresponding cyclic voltammograms are provided in the Supporting Information (Figure S3).

Both PANI-MOF composites show much higher cycling stability compared to pristine PANI. Figure 12 shows that after 1000 cycles, the gravimetric capacitance of pristine PANI decreases to 77% of the initial value. At the same time, the residual capacitance of PANI-UiO-66 and PANI-UiO-66-NH_2_ does not decrease below ≈100%. The initial cycles for the composites show an increase in capacitance, which is likely attributed to the improvement of the electrode material wetting with the electrolyte. These results show that, unlike pristine PANI, the prepared composites are attractive electrode materials that can withstand multiple charge–discharge cycles in energy applications.

## Conclusions

Composites of PANI and Zr-based MOFs, UiO-66 and UiO-66-NH_2_, have been successfully prepared by the oxidative polymerization of aniline. It has been shown for the first time that using two similar MOF types, different in terms of the presence of amino groups, for the composite preparation affects aniline polymerization and the type of PANI binding to them, which can be covalent or noncovalent. The direct evidence for the covalent binding of PANI to amino groups of UiO-66-NH_2_ was provided by solid-state NMR and supported by vibrational spectroscopy. The nature and chemical functionalization of the MOF, affecting the PANI interaction with it, also directly determines the electrochemical behavior of the composites as shown by cyclic voltammetry. The enhanced cycling stability can be potentially useful in energy storage applications.

## Supporting Information Available

Full-range experimental ^13^C CP/MAS NMR spectra; cyclic voltammogram of pristine PANI; gravimetric capacitance of materials measured at various scan rates; and cyclic voltammograms corresponding to cycling stability studies (PDF)
