# Supplementary material for: Effect of a Zr-Based Metal–Organic Framework Structure on the Properties of Its Composite with Polyaniline
Source: ACS Appl Mater Interfaces. 2023 May 4;15(19):23813–23. doi: 10.1021/acsami.3c03870 (PMC10197080; doi:10.1021/acsami.3c03870)
Supplement: Supplementary file 1 — am3c03870_si_001.pdf [file am3c03870_si_001.pdf]

Supporting Information for:

Effect of Zr-based metal-organic framework structure on the properties of its composite with polyaniline

Konstantin A. Milakin[1], Sonal Gupta[1], Libor Kobera[1], Andrii Mahun[1,2], Magdalena Konefał[1], Olga Kočková[1], Oumayma Taboubi[1], Zuzana Morávková[1], Jia Min Chin[3], Kamal Allahyarli[3], Patrycja Bober[1*]

[1]Institute of Macromolecular Chemistry, Czech Academy of Sciences, 162 00 Prague, Czech Republic

[2]Department of Physical and Macromolecular Chemistry, Faculty of Science, Charles University, 128 40 Prague, Czech Republic

[3]Institute of Inorganic Chemistry-Functional Materials, University of Vienna, A-1090 Vienna, Austria

[*]Corresponding author e-mail address: bober@imc.cas.cz (Patrycja Bober)

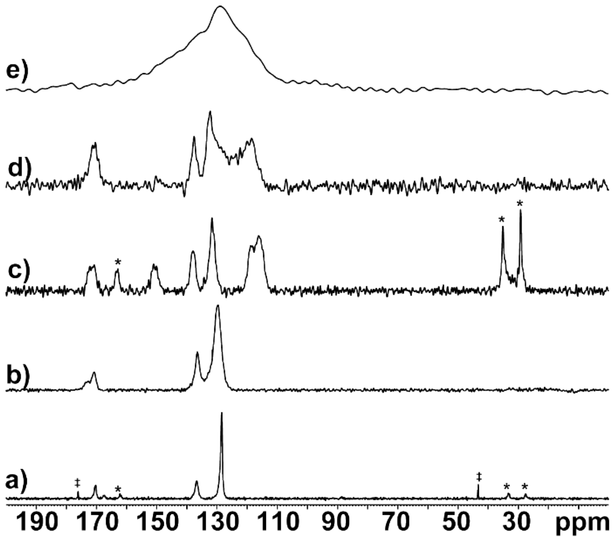

Figure S1. Full range experimental ^{13}C CP/MAS NMR spectra of a) UiO-66, b) PANI-UiO-66, c) UiO-66-NH_2, d) PANI-UiO-66-NH_2 and ^{13}C VF/MAS NMR of e) PANI.

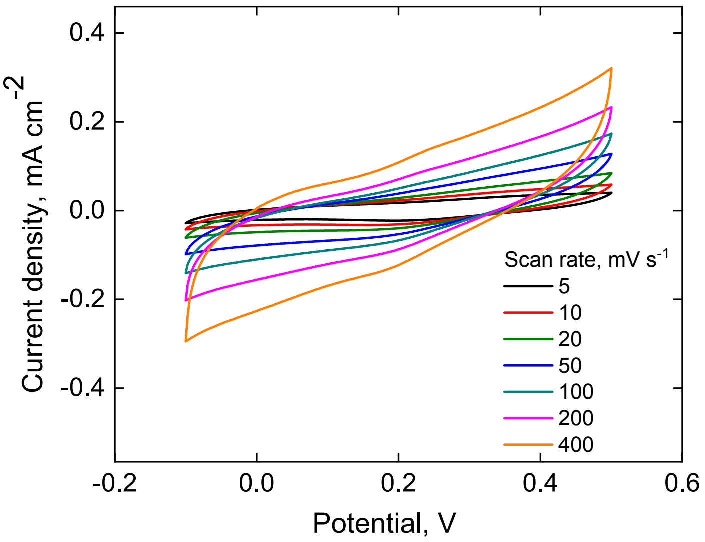

Figure S2. Cyclic voltammogram of pristine PANI, recorded at various scan rates in 0.01 M HCl.

Table S1. Gravimetric capacitance of PANI-UiO-66-NH_2, PANI-UiO-66 and pristine PANI, normalized per mass of the active material, measured at various scan rates.

**Potential scan rate, mV s^{-1}**	**Gravimetric capacitance (per PANI mass), F g^{-1}**		
	PANI-UiO-66	PANI-UiO-66-NH_2	PANI
**5**	33.0	79.8	50.5
**10**	16.5	48.4	35.6
**20**	10.6	38.7	24.4
**50**	9.2	41.1	14.4
**100**	9.6	47.6	9.6
**200**	11.5	54.0	6.5
**400**	13.8	56.4	4.6

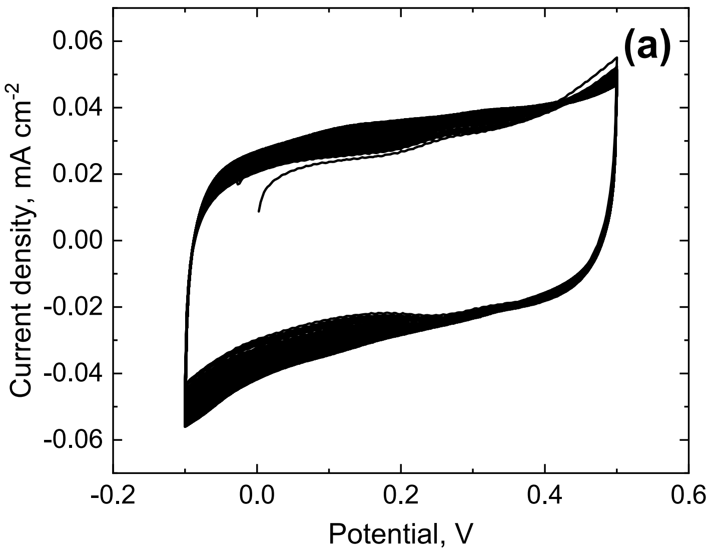
(a)
0.06
0.04
0.02
0.00
-0.02
-0.04
-0.06
Current density, mA cm-2
-0.2
0.0
0.2
0.4
0.6
Potential, V

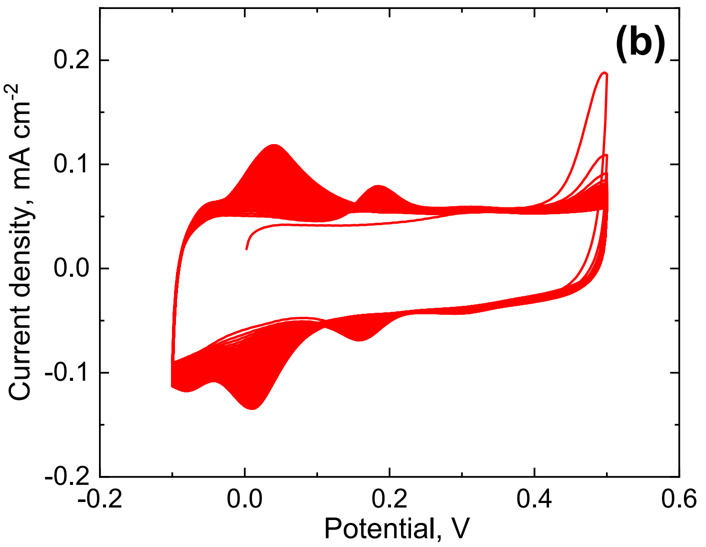
(b)
0.2
0.1
0.0
-0.1
-0.2
Current density, mA cm-2
-0.2
0.0
0.2
0.4
0.6
Potential, V

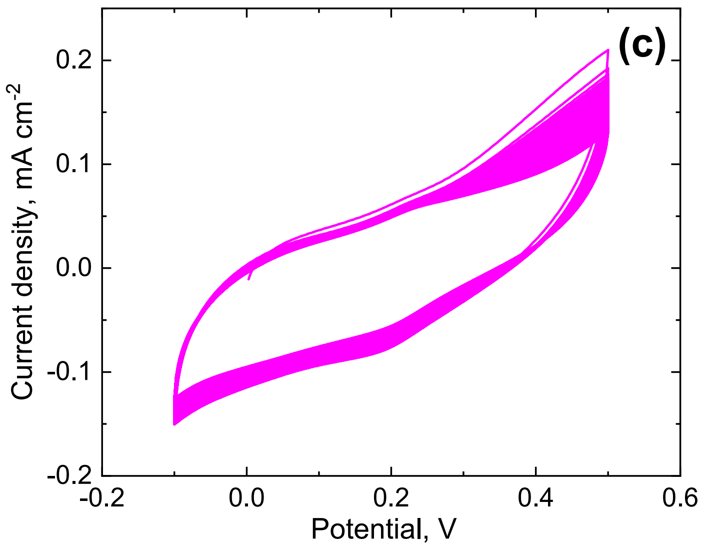

Figure S3. Cyclic voltammograms of a) PANI-UiO-66, b) PANI-UiO-66-NH_2 and c) PANI, recorded for 1000 cycles at 100 mV s^{-1} in 0.01 M HCl.
